# Safety and potential efficacy of gemfibrozil as a supportive treatment for children with late infantile neuronal ceroid lipofuscinosis and other lipid storage disorders

**DOI:** 10.1186/s13023-017-0663-8

**Published:** 2017-06-17

**Authors:** Kyeongsoon Kim, Hynda K. Kleinman, Hahn-Jun Lee, Kalipada Pahan

**Affiliations:** 1 Department of Pharmaceutical Engineering, Inje University, Gimhae, South Korea; 2 Polaryx Therapeutics Inc., Paramus, NJ USA; 3 The George Washington University Medical Center, Washington, DC USA; 4 Rush University Medical Center, Chicago, IL USA

**Keywords:** Batten disease, NCL, Gemfibrozil, Lipofuscinosis, Central nervous system, Lysosome biogenesis, TPP1, Lopid, CLN2, Children

## Abstract

Neuronal Ceroid Lipofuscinosis (NCL), also known as Batten disease, is a group of genetically distinct lysosomal disorders that mainly affect the central nervous system, resulting in progressive motor and cognitive decline primarily in children. Multiple distinct genes involved in the metabolism of lipids have been identified to date with various mutations in this family of diseases. There is no cure for these diseases but some new therapeutic approaches have been tested that offer more hope than the standard palliative care. Many of the therapeutic advances require invasive procedures but some progress in slowing the disease has been found and more options can be expected in the future. We also review the literature on children with disease/conditions other than NCL for the non-invasive use, safety, and tolerability of a lipid-lowering drug, gemfibrozil, as a potential treatment for NCLs. Gemfibrozil has shown efficacy in an animal model of NCL known as CLN2 (late infantile classic juvenile) and has been shown to be safe for lowering lipids in children. Among the 200 non-NCL children found in the published literature who were treated with gemfibrozil for NCL-related problems, only 3 experienced adverse events, including 2 with muscle pain and 1 with localized linear IgA bullous dermatitis. We conclude that gemfibrozil is safe for long-term use in children, causes minimal adverse events, is well tolerated, and may delay the progression of NCLs. Gemfibrozil may potentially be an alternative to more invasive therapeutic approaches currently under investigation and has the potential to be used in combination with other therapeutic approaches.

## Background

Neuronal Ceroid Lipofuscinsosis (NCL), also known as Batten disease, is a group of inherited neurodegenerative diseases affecting approximately 1–4 children per 100,000 live births [1–4]. NCLs mainly affect children and generally start with seizures and/or a loss of vision at an early age followed by rapid motor and cognitive decline leading to premature death.

NCLs are generally autosomal recessive, and the frequency of these diseases varies by genetic mutation and by country. To date, over 400 different mutations are found in multiple genes [2, 3, 5]. A data-base contains information on the specific mutations identified to date (http://www.ucl.ac.uk/ncl).

Most of the proteins encoded by the mutated genes are lysosomal, including soluble enzymes (CLN1/PPT1, CLN2/TPP1, CLN10/CTSD, CLN13/CTSF) or a soluble lysosomal protein (CLN5) [2]. One protein is also present in the secretory pathway (CLN11). In addition, there are trans membrane proteins (CLN3, CLN6, CLN7/MFSD8, CLN8, CLN12/ATP132A) and two cytoplasmic proteins that associate peripherally with membranes (CLN4, CLN14). Two trans membrane proteins (CLN6 and CLN8) localize in the endoplasmic reticulum while others (CLN4/DNAJC5 and CLN14/KCTD7) are cytoplasmic and associate with cell membranes.

The functional substrates of these proteins are not known, and the major functions of the trans membrane proteins in general and in the disease etiology are unclear [6]. The age of onset, severity, and rapidity of progression differ for each of the mutations in these genes and for the different mutations within each gene [2]. Thus, a complex family of related lipid storage diseases with different genetic etiologies forms the NCL family. Such diversity in the genes and in the gene mutations can make finding a comprehensive therapeutic treatment challenging for the largely unknown and complex pathophysiology of the NCLs.

## Therapeutic approaches for NCLs

There is no cure for NCLs at this time. The standard of care is generally to minimize symptoms, especially seizures. Many new therapies are emerging and hopefully some will halt or slow disease progression but none are likely to reverse the damage [7–9]. Based on a systematic search of the literature and clinicaltrials.gov, several approaches offer promising therapeutic strategies, including gene therapy, stem cell therapy, enzyme replacement therapy, anti-inflammatories, and small molecules. All are being tested on animal models and in some cases the studies have progressed to early stage human trials. Natural and genetically modified mice and other animal models, including large animals, such as pigs, sheep, cows, and dogs, have greatly helped in determining delivery mode, ability to cross the blood-brain-barrier, dosing amount, frequency of dosing, and efficacy. Unfortunately at this time, the work is primarily experimental and invasive in many cases but proof of concept and improved treatment regimens offer considerable hope. The complexity and number of genes and mutations affected is problematic in developing and testing new treatments. Below are some examples of the types of therapeutic approaches and their success to date. One can expect many more treatment options with additional emerging technologies.

Viral-mediated gene therapy for NCLs generally involves the use of a viral vector carrying a normal form of the mutated gene localizing in the central nervous system [10–12]. When the virus enters the cells in the brain, the cells then express normal copies of the mutated protein that are taken up by the nearby cells. This approach is attractive because it offers the potential for a lifelong cure that does not require multiple repeated treatments. It therefore is less invasive for the patient than other approaches. There are many additional advantages to viral vectors, including their tropism for certain cells. However, these vectors, particularly the adeno-associated viruses, do induce an immune response. Lentiviral vectors have a reduced immune response but their integration can be non-specific and is limited to the site of injection [13]. One concern is the lack of potential therapeutic efficacy for the lipid accumulation in organs outside of the brain if the vector is delivered to the brain. Using TPP1-deficient dogs treated with an adenoviral vector containing TPP1, it was found that the protein was increased in both the heart and spleen but not in the kidney or liver [14]. Additional studies are needed to optimize the efficacy and reduce the potential immune response issues for viral vectors. Various animal models have been tested with adeno-associated viral vectors and increased survival along with improved motor skills were observed [12]. Based on these successful results, phase 1 safety trials (NCT 01161576, NCT01414985, and NCT00151216) of an adeno-associated viral gene transfer vector for CLN2 in the brain of children with late infantile NCL are ongoing, and 1 phase 1/2a (NCT02725580) for CLN6 with scAAV9 has begun.

Enzyme replacement therapy involves preparing a large amount of protein based on the normal version of the mutated gene usually by recombinant methods and injecting the recombinant protein (rhTPP1) into the tissues, namely the brain for CNL2 [15–21]. The disadvantages are that the blood-brain-barrier has to be considered and there is a need for repeated injections. Mice, dogs, and monkeys tested with various doses and different routes of administration have provided positive results and led to a clinical trial for late infantile NCL (NCT01907087) that is now complete. It is exciting that this trial has reported positive results for CLN2 protein replacement and that the treatment with recombinant TPP1 (also known as cerliponase alpha or Brineura) was recently approved by the FDA. The treated patients showed a significant efficacy in the disease rating scale relative to that of natural history controls i.e. disease progression was reduced in the treated children. The disadvantages are that for this level of success, the patients had 1 site in their brain injected every other week for 48 weeks. The procedure is not a cure and is invasive and costly. Enzyme replacement therapy is likely to work for the patients with the mutated and soluble lysosomal proteins (CLN1/PPT1, CLN2/TPP1, CLN10/CTSD, CLN13/CTSF, CLN5) but not for the patients with mutated trans membrane and membrane-associated proteins (CLN3, CLN4/DNA JC5, CLN 5, CLN6, CLN7/MFSD8, CLN8, CLN14) [4]. Further studies on the dosing amount and frequency, penetration through the blood-brain-barrier, etc. are needed for optimization of enzyme replacement therapy for NCLs.

Small molecule therapy involves enhancing activity by correcting signaling or cellular metabolic pathways and includes drugs that may act as pharmacological chaperones, receptor modulators, and immune modulators or reduce the amount of substrate [7, 8, 22, 23]. Chaperones have the advantage that they are small, can distribute widely, and can be taken orally [22, 23]. The disadvantage is that such drugs will have limited use as they act only on proteins where the mutation is not near the active site but has caused a protein-folding problem. The use of receptor modulators is potentially a powerful approach, and improvement in the motor function of a mouse model of CLN3 has been observed with an AMPA antagonist [7, 24–26]. However, substrate reduction therapy with Cystagon has not been successful (NCT00028262) in reducing disease progression [27]. Immune modulators are a logical approach as inflammation precedes the neurodegeneration observed in NCLs. Several immune modulators have been tested without success [28, 29]. A recent phase 2 trial (NCT01399047) for CLN3 with mycophenolate given for 8 weeks has yet to report the results. These small molecule therapies will likely become relevant as advances are made in delivery and in their use in other neurodegenerative diseases.

Stem cell therapy, including bone marrow cells, is aimed at having the stem cells incorporate into tissues and provide a source of non-mutated proteins. It is also possible that the stem cells will regenerate the tissue that has been damaged or lost. Stem cell therapy is thus attractive because it offers the potential to replace damaged or lost tissue in advanced patients. To date, hematopoietic stem cell transplantation has shown no efficacy in NCL patients [30, 31]. Neural stem cells have also been tested in a mouse model of CLN2 and some reduction in the storage of lipids was found [32]. Fetal neural stem cells have been injected into two locations in the brain as part of a safety and efficacy trial in patients with advanced disease (NCT00337636). Immunosuppression was used to reduce complications. The results of the trial showed good tolerability and an increased production of CLN2 enzyme but additional studies are needed to optimize this approach for reducing or reversing disease progression.

In summary, there are many different types of approaches being used to develop therapeutics for the NCL family of diseases. These studies have been aided greatly by the use of small and large animal models of the different diseases [33, 34]. The exciting success of enzyme replacement therapy for CLN2 is a proof of principal that replacing the mutated soluble and secreted proteins will be effective and offers the families and children a treatment option. Marked progress is being made with other treatment approaches, such as gene therapy and stem cells, as well as with small molecules [8]. Furthermore, new technologies, such as gene editing and new classes of treatments, are emerging that may advance some of these treatments. Because of the complex genetics of the NCLs, these existing approaches may be limited in applicability to each condition. The devastating effects on young children are rapid, often not immediately diagnosed so that the disease has progressed at the time of diagnosis in many cases, and the invasive nature of the majority of these treatments can deter the families from opting to try them. Recently, a simple and safe lipid lowering drug, gemfibrozil, has shown efficacy in cells and in an animal model and may offer the patients a non-invasive option for treatment either alone or in combination with other emerging treatments [35–49].

## Gemfibrozil (Lopid)

**Fig. 1:**
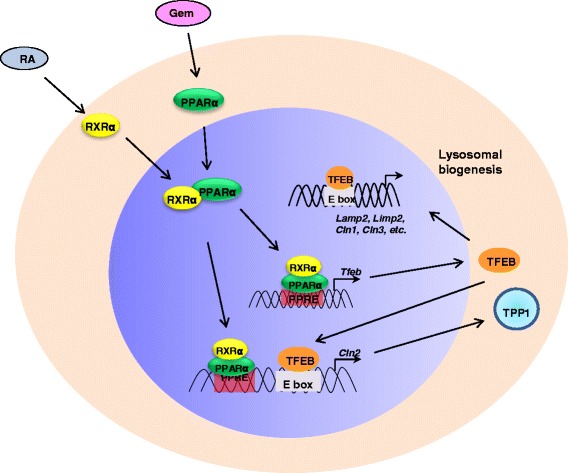
Scheme showing the dual mechanism of action of gemfibrozil

Gemfibrozil is a member of the fibrates group of drugs that lower lipid levels [35]. It was originally shown to lower lipids in animals in the 1960s and was approved by the FDA in 1976 for human use to reduce serum lipids. It reduces the levels of triglycerides, very low-density lipoprotein (VLDL, “bad cholesterol”), and low-density lipoprotein (LDL, “bad cholesterol”) and increases high-density lipoprotein (HDL, “good cholesterol”). Gemfibrozil is an activator of peroxisome proliferator-activated receptor-alpha (PPARα), a nuclear receptor important in the metabolism of fats (Fig. 1) [36–40]. It also regulates adipose tissue differentiation. It thus promotes the clearance of lipids [35]. A major advantage is that it is taken orally as a 600 mg tablet twice a day before meals and has minimal side effects. In fact, gemfibrozil has been found to have many additional effects on reducing inflammation, regulating oxidative stress, promoting signal transduction, increasing myelination, etc. [40, 41] which have led to many past and ongoing clinical trials in other disease pathologies (www.clinicaltrials.gov).

## The mechanism of action of Gemfibrozil on lowering lipids

Fatty acids are metabolized in mitochondria and in peroxisomes. While short, medium, and long chain fatty acids are metabolized in mitochondria, very long chain fatty acids (VLCFAs) are chain-shortened in peroxisomes via peroxisomal β-oxidation. Gemfibrozil being one of the prototype activators of PPARα stimulates peroxisomal β-oxidation by up-regulating the expression of all three important peroxisomal β-oxidation enzymes (acyl-CoA oxidase, 2-*trans*-enoyl-CoA hydratase, and thiolase) via PPAR-α (Fig. 1) [39, 41]. Since VLCFAs are major components of LDL and VLDL, a gemfibrozil-mediated decrease in bad cholesterol (VLDL and LDL) is directly correlated to increased catabolism of VLCFAs in peroxisomes.

## How does gemfibrozil modulate TPP1?

Gemfibrozil acts in two ways to increase TPP1 in cells (Fig. 1). Gemfibrozil activates PPARα, which enhances the mRNA and protein levels of transcription factor EB (TFEB) by more than 10-fold and 6-fold, respectively, in brain cells within 12 h [39]. TFEB then binds to the promoter of genes involved in lysosome biogenesis and activates their production [43–46]. TFEB can regulate lysosomes due to its effects on the expression on lysosomal genes. All-trans retinoic acid has similar activity. The combination of all-trans retinoic acid with gemfibrozil also enhances TFEB production within 12 h, and lower doses of both compounds provided maximal activity in enhancing lysosome biogenesis in brain cells and in cells derived from patients with CLN2/TPP1 [38]. Gemfibrozil also binds to the retinoid X receptor-α (RXRα), which binds to PPARα thereby up-regulating the expression of TPP1 in brain cells via the PPARα/RXRα heterodimer. Increased TPP1 activity has been observed in a variety of neuronal cells and fibroblasts treated with gemfibrozil but not in iPSC cells (Table 1) [38, 47]. Based on these dual activities, gemfibrozil has potential for therapeutic applications in the NCLs.

**Table 1:** Effect of gemfibrozil on cells and on animals

	Treatment	Effect	Reference
*Cells* in vitro			
Mouse & human primary astrocytes	gemfibrozi 25 μm	↑CLN2 mRNA ↑TPP1 protein	38, 39
Mouse neurons from cortex, striatum & hippocampus	gemfibrozil 25 μm	↑TPP1 protein	38
Lymphoblastic cells from CLN3 patients	gemfibrozil 25 μm	↑viability ↑autophagy recovery ↑autophagy genes	48
Human IPS models CLN2 and CLN3 mutations	gemfibrozil 25 ﻿µ﻿m	No effect on TPP1	47
Animals			
Mouse	oral gemfibrozil 7.5 mg/kg for 21 d	↑TTP1 in astrocytes, cortical neurons, & non neural cells of dentate gyrus & CAI of hippocampus	38
Mouse KO LINCL	oral gemfibrozil 7.5 mg/kg for 21 d	↑longevity, ↑motor retention, ↓apoptosis, ↑anti-inflammatory molecules	49

d = days

## Studies on cells treated with gemfibrozil

Various cells in culture have been treated with gemfibrozil (38,47,48) (Table 1). The effect of gemfibrozil on *Cln2* mRNA, protein expression, and TPP1 activity has been studied in primary mouse neurons and in astrocytes and in human astrocytes, and neuronal cells from different regions of the brain (cortex, hippocampus, striatum, and cerebellum). Gemfibrozil increased the mRNA for *Cln2* with a maximum increase at 24 h in mouse primary astrocytes [38, 39]. Interestingly the mRNA for other lysosomal genes, such as *Cln1* and *Cln3,* were also increased by gemfibrozil. Increased TPP1 protein was observed inside the cells as well. Neuronal cells and human astrocytes also showed a similar response with increased mRNA and protein that was validated by either western blot, immunofluorescence, and/or by a TPP1 activity assay [38]. The effect of gemfibrozil on *TPP1* mRNA was evaluated in wild type, PPARα deficient, and PPARβ deficient astrocytes isolated from cells from wild type and genetically modified mice [39]. Interestingly, gemfibrozil did not increase *Cln2* mRNA levels in the cells from the PPARα knock out (KO) mice whereas the increase in *Cln2* mRNA was seen with gemfibrozil treatment of cells from both wild type and PPARβ KO mice. Immunofluorescence, western blot, and an enzyme activity assay confirmed the lack of *Cln2* mRNA up-regulation by gemfibrozil in the cells isolated from the PPARα KO mice [38]. Similar studies were done with siRNA-treated cells where there was no increase in TPP1 with gemfibrozil, and this study further demonstrated the role of RXRα in regulating the expression of *TPP1* mRNA. These studies confirm the involvement of PPARα/RXRα in the gemfibrozil-mediated increase in both *Cln2* mRNA and TPP1 protein [38]. It was concluded that gemfibrozil and retinoic acid recruit both PPARα and RXRα to the RXR-binding site of the *Cln2* gene promoter.

Some studies have been done on cells derived from NCL patients and models (Table 1) [48, 49]. Lymphoblasts isolated from control and NCL patients were investigated for the effect of gemfibrozil on apoptosis, depolarization of the mitochondrial membrane, and defective autophagy [49]. NCL (CLN3) lymphoblast cell viability to normal levels was restored by gemfibrozil in NCL patient-derived lymphoblasts via decreased apoptosis. Furthermore, the high level of membrane potential depolarization of NCL patient-derived lymphoblasts was restored to normal levels by gemfibrozil, and defective autophagy was normalized by gemfibrozil. These findings suggest that gemfibrozil may have a therapeutic effect by multiple mechanisms for NCL patients [39, 40].

## Studies on animals treated with gemfibrozil

**Fig. 2:**
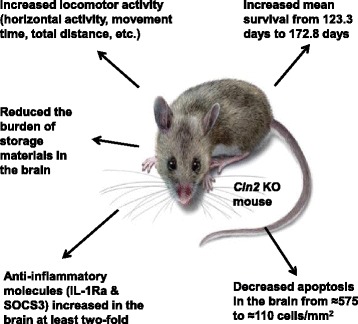
Summary of the effects of gemfibrozil on the CLN2 knock out mouse

The effect of gemfibrozil has been evaluated in a newly created mouse model of CLN2/TPP1 of NCL (Figure 2) [49]. This model was created by knocking out the gene for TPP1. The mice develop progressive motor dysfunction and die prematurely. A dose of 7.5 mg/kg body weight of gemfibrozil increased survival by several weeks to 172 days over that observed with vehicle alone, 123 days. Furthermore, the mice showed improved motor functions over the controls. Both SOCS3 and IL-Rα are anti-inflammatory factors that are increased with gemfibozil treatment. The anti-apoptotic molecule phospho-Bad also increased and was coincident with decreased neuronal loss based on a TUNEL assay. Finally, a decrease in storage materials in the motor cortex of the brain was observed in the gemfibrozil-treated *cln2* knock out mice as compared with the untreated knock out mice [49]. Interestingly, since these genetically-modified mice lack the *cln2* gene, gemfibrozil is likely not working by increasing TPP1 expression but rather via lysosome biogenesis and other activities. The findings suggest that gemfibrozil can delay the progression of neurodegenerative decline in an animal model by multiple mechanisms. Clearly optimizing the dose and frequency of dosing may further improve the outcome with the animals. The data also suggest that the neuroprotection by gemfibrozil may be applicable to treating children with CLN2/TPP1 NCL and possible other genetic variants of the NCLs [48, 49].

When wild type and PPARα and PPARβ KO mice were analyzed for the effect of gemfibrozil on TPP1 expression, gemfibrozil markedly increased TTP1 protein expression in the cells of the brains of the wild type and PPARβ KO mice but not in the PPARα KO mice [38, 39]. These findings again demonstrate the involvement of PPARα in the gemfibrozil-mediated increase in TTP1 (38). Furthermore, the *Cln2* gene contains an RXR-binding site, and all-trans retinoic alone can increase TTP1 expression. The studies further confirm the mechanism of action of gemfibrozil in vivo and demonstrate the role of the PPARα/RXRα heterodimer in its activity.

## Safety of gemfibrozil: Studies with children treated with gemfibrozil

Having been approved for human use in 1976, gemfibrozil has a long history of safety and tolerability in adults as a lipid-lowering agent. It has also been used with children for various indications (Table 2). In a small study with 12 children aged 5–17 years who had hyperlipidemia with persistent nephrotic syndrome, gemfibrozil given for 4 months to 7 of the patients (5 were controls) was found to be effective in improving the lipoprotein profile of the nephrotic children [50]. Besides efficacy, gemfibrozil had no side effects, and renal function and urine protein excretion in this fragile population were not affected by the treatment. Gemfibrozil was also given in 2 doses 12 h apart in a study with 97 neonates (49 treated and 48 control) of at least 34-week gestation with non-hemolytic jaundice. While there was no change in the need and duration of phototherapy with the gemfibrozil, it was noted that there were no side effects in the preterm and term neonates [51]. In another pilot study on 47 pediatric patients with metabolic syndrome, a dose of 1200 mg/day gemfibrozil for 8 months significantly lowered the triglyceride levels and raised the HDL levels [52]. Two of the patients had muscle pain associated with the treatment but no other safety issues were found. Muscle pain was reported as a probable adverse event in adults. Further, it has been suggested that fibrates should be considered for children with obesity-related hyperlipidemias [53]. Two siblings with familial chylomicronemia syndrome were treated from birth (oldest child age 7 in the study) and at 6 months of age (younger child age 4 in the study) with 300 mg gemfibrozil twice per day [54]. At 7 and 3.5 years respectively, there were no side effects from the gemfibrozil, and the risk of acute pancreatitis, a complication of hyperlipidemia, was significantly reduced. There was one case report of an age 13 female patient who developed linear IgA bullous dermatitis on her areola after 3 weeks on gemfibrozil, which resolved with drug withdrawal and steroid treatment [55]. Although side effects have been reported in adults taking gemfibrozil (Table 3), these are generally uncommon and rarely severe.

**Table 2:** Safety of gemfibrozil in children

Age	Dose (duration)	Purpose	Effect	Side effects (reference)
Children with metabolic Syndrome, *n* = 47	600 mg 2 x /day (8 months)	reduce lipids	↓triglycerides ↑HDL	1AE muscle pain *n* = 2 (52)
Late preterm & term neonates with jaundice *n* = 97 (49 active, 48 placebo)	60 mg/kg 2 doses (1 day)	reduce light therapy time, decrease bilirubin	no effect	none (51)
Infants with Chylomicronemia* Syndrome, *n* = 2	300 mg 2 x /day (3, 7 years)	improve jaundice	↓pancreatitis	none (54)
Children with hyperlipidemia & nephrotic syndrome, *n*=12 (7 active, 5 placebo)	150 mg 2 x/day (4 months)	reduce lipids	↓cholesterol ↓triglycerides	none (50)
Age 13 female *n* = 1	3 weeks	reduce lipids	NA	LABD (55)

*Siblings started gemfibrozil at birth and at 6 months, respectively

*LABD*, linear IgA bullous dermatitis

**Table 3:** Events related to treatment with gemfibrozil

	Causal relationship	Causal relationship
	Probable	Not established
General:	weight loss	
Cardiac	extrasystoles	
Gastrointestinal	cholestatic jaundice	pancreatitis
		hepatoma
		colitis
Central Nervous System	dizziness	confusion
	Somnolence	convulsions
	Paresthesia	syncope
	peripheral neuritis	
	decreased libido	
	depression	
	headache	
Eye	blurred vision	retina edema
Genitourinary	impotence	decreased male fertility
		renal dysfunction
Musculoskeletal	myopathy	
	myasthenia	
	myalgia	
	painful extremities	
	arthralgia	
	synovitis	
	rhabdomyolysis	
Clinical Laboratories	increased creatine	positive antinuclear antibody
	Phosphokinase	
	increased bilirubin	
	increased liver	
	transaminases (AST, ALT)	
	increased alkaline	
	phosphatase	
Hematopoietic	anemia	thrombocytopenia
	leukopenia	
	bone marrow hypoplasia	
	eosinophilia	
Immunologic	angioedema	anaphylaxis
	laryngeal edema	Lupus-like syndrome
	urticarial	vasculitis
Integumentary	exfoliative dermatitis	alopecia
	Rash	photosensitivity
	dermatitis	
	pruritus	

SPC (Standard of Product Characteristics) of LOPID® (Gemfibrozil Tablets, USP) by Pfizer, Revised March 2016

These studies demonstrate that gemfibrozil was well tolerated and was safe for use in children even with ongoing disease processes. Based on the known drug interactions of gemfibrozil (Table 4), children with NCL should not be expected to experience problems. However, with gemfibrozil treatment there may be a potential reduction in the effectiveness of the anti-convulsive therapies that should be monitored. The off label use of gemfibrozil in ill children over a range of ages, doses, and durations suggests that gemfibrozil can be expected to be safe for use in children with lipid storage diseases.

**Table 4:** Drug interactions with gemfibrozil

Concomitant medication	Cautions
HMG-CoA Reductase Inhibitors	risk of myopathy and rhabdomyolysis
Anticoagulants	warfarin dosage should be reduced
CYP2C8 Substrates	drugs metabolized CYP2C8 (e.g., dabrafenib, loperamide, montelukast, paclitaxel, pioglitazone, rosiglitazone) may be required to reduce
OATP1B1 substrates	substrates of OATP1B1 (e.g., atrasentan, atorvastatin, bosentan, ezetimibe, fluvastatin, glyburide, SN-38 [active metabolite of irinotecan], rosuvastatin, pitavastatin, pravastatin, rifampin, valsartan, olmesartan) may be required to reduce
Bile Acid-Binding Resins	resin-granule drugs such as colestipol (5 g) are recommended at 2 or more hours apart
Colchicine	myopathy, including rhabdomyolysis in chronic administration of colchicine

SPC of LOPID issued March.2016

## Summary and conclusions

There are several advantages for use of gemfibrozil in children with lysosomal storage diseases, NCLs, and in particular late infantile batten disease CLN2/TPP1. Gemfibrozil is an oral drug that is not invasive and has few side effects in adults and in children. Gemfibrozil has multiple modes of action, and the mechanisms of action are known for some of the activities, such as its ability to increase TPP1 mRNA, protein, and activity, reduce inflammation, increase myelination, and increase lysosome biogenesis [39, 40]. Gemfibrozil can also affect various signaling pathways involved in switching off T-helper cells, cell-to-cell contact, migration, apoptosis, oxidative stress, and inflammation. Finally, using knock out animal models of CLN2/TPP1, gemfibrozil was found to prolong the lifespan of the animals, decrease lipid accumulation in the motor cortex, delay the loss of mobility, and increase certain genes involved in anti-inflammation and in anti-apoptosis [49]. This neuroprotective effects combined with the potential increases in TPP1 enzyme levels and in biogenesis of lysosomes suggest that gemfibrozil will have some benefit in treating early stage children with CLN2/TPP1 and possibly with other genetic forms of NCLs. The oral administration is particularly attractive because it is non-invasive and the safety is well documented. Gemfibrozil could be used alone or combination with some of the emerging treatments for these devastating genetic diseases in children. One possible limitation might be weight loss that will need to be monitored since NCL patients have weight gain issues.

## Abbreviations

HDL: High density lipoprotein
LCFA; LDL: low density lipoprotein NCL Neuronal Ceroid Lipofuscinosis
PPAR: Peroxisome proliferator-activated receptor
PPT1: Palmitoyl-protein thioesterase 1
RXR: Retinoid X receptor
TFEB: Transcription factor EB
TPP1: Tripeptidyl peptidase 1
VLCFA: Very long chain fatty acids
VLDL: Very low density lipoprotein
